# “Trust and suspicion” Client and provider perspectives on the acceptability of medication for opioid use disorder among people who inject drugs in Kampala, Uganda

**DOI:** 10.21203/rs.3.rs-6819472/v1

**Published:** 2025-06-17

**Authors:** Peter Mudiope, Nicholas Nanyeenya, Okurut Simon, Adelline Twimukye, Kibira Simon, Mutamba Byamah Brian, Joan Nangendo, Stella Alamo, Fredrick Makumbi, Rhoda Wanyenze, Joseph KB Matovu

**Affiliations:** Makerere University School of Public Health; Makerere University School of Public Health; Makerere University School of Public Health; Makerere University; Makerere University School of Public Health; Butabika National Referral Mental Hospital; Makerere University College of Health Sciences; United States of America Centres for Disease Control and Prevention; Makerere University School of Public Health; Makerere University School of Public Health; Makerere University School of Public Health

**Keywords:** MOUD, acceptability, people who inject drugs, Uganda

## Abstract

**BACKGROUND:**

Despite strong evidence supporting medication for opioid use disorder (MOUD), acceptability varies considerably across contexts. This study explored client and provider perspectives on MOUD acceptability among people who inject drugs (PWID) in Kampala, Uganda.

**METHODS:**

We conducted a qualitative descriptive study during November and December 2023 in Kampala Capital City, Uganda. In-depth interviews with 20 PWID (10 enrolled, 10 not enrolled) and key informant interviews with 10 MOUD service providers were conducted. Data were analysed using thematic analysis, guided by the Theoretical Framework of Acceptability (TFA) and managed with ATLAS.ti software.

**RESULTS:**

MOUD was highly acceptable among providers, enrolled and non-enrolled participants. Facilitators included comprehensive, person-centered services that addressed health and psychosocial needs, supportive family relationships, alignment with personal recovery goals, and the safety and effectiveness of supervised medication therapy. However, participants encountered significant barriers. Structural challenges such as high transport costs, limited clinic operating hours, and strict enrolment criteria impeded access and continuity. Fear of arrest due to drug criminalization and stigma, both societal and within healthcare settings, further discouraged engagement. Additionally, some participants questioned methadone’s effectiveness relative to heroin and reported widespread reliance on traditional and spiritual healing practices, often coerced by family members. Social norms promoting mutual drug-sharing as a symbol of trust were disrupted by MOUD enrolment, resulting in peer resistance and social isolation.

**CONCLUSION:**

MOUD was highly acceptable among PWID and providers in Uganda, valued for its effectiveness in restoring health and stability. However, structural barriers, stigma, misinformation, and restrictive enrolment often drove PWID to non-evidence-based treatment. Flexible service models, simplified enrolment procedures, community sensitisation, and collaboration with abstinence-based providers and law enforcement are vital to improving MOUD acceptability.

## BACKGROUND

The opioid crisis affects millions of people and strains healthcare systems globally. According to the UNODC World Drug Report 2023, approximately 13.2 million people globally injected drugs in 2021, marking an 18% increase from the 11.2 million estimated in 2020 [1]. The act of injecting drugs leads to multiple health and socio-economic impacts, including HIV infection and Hepatitis B & C, along with higher rates of overdose-related death and unemployment [1]. Globally, medication for opioid use disorder (MOUD) is recommended as an effective treatment for reducing drug and sex-related risk of HIV, hepatitis and other blood-borne infection transmission among people who inject drugs (PWID) [2]. Previous studies show that MOUD improves HIV treatment retention and outcomes, quality of life, and reduces mortality [2–5]. In MOUD programs, PWID are offered medications like Methadone, buprenorphine, and naltrexone to reduce opioid cravings, withdrawal symptoms, and relapse. In addition, risk reduction counselling, education, and psychosocial support are administered to PWID as part of treatment [2].

Despite the proven efficacy, the acceptability and uptake of MOUD programs vary considerably across different populations and regions [1, 6]. Obstacles to the uptake of MOUD services include stigma and discrimination, social and cultural beliefs, limited access to healthcare services, and inadequate knowledge about MOUD. Economic constraints, such as lack of income, unstable housing, legal and policy restrictions in countries where drug use is illegal, further hinder acceptability. Because of these barriers, patients with substance use disorders and mental illness may resort to seeking treatment from traditional or complementary medicine practitioners [7–9]. Research into patient preferences demonstrated that over 45% prefer traditional medicine practitioners [10]. Traditional healers provide affordable services tailored to individual needs while integrating with cultural systems and eliminating stigma [11, 12]. The lack of a robust system to support and regulate traditional medicine means that patients who opt for these practitioners face delays when they need evidence-based treatments.

By December 2023, the only MOUD clinic in Uganda had enrolled over 490 clients since its inception in 2020, with annual retention of about 55% [13]. The MOUD provides distinct benefits, but operates under strict rules and regulations. Clients must undergo a thorough preparation process before enrolment, which includes a comprehensive check before they enter the clinic premises, and they face punishment for missing four consecutive days of clinic attendance. The responses of injection drug users (IDU) to the clinic’s “dos and don’ts” vary when influenced by different socio-cultural factors and partially explain the clinic’s decreasing enrolment and retention rates[13]. The study examined both conceptual and operational factors influencing the acceptability of medication for opioid use disorder, to generate evidence for enhancing the MOUD effectiveness program in Uganda.

## METHODS

### Study design

From November to December 2023, we conducted a qualitative descriptive study employing in-depth face-to-face interviews with individuals who inject drugs and key informant interviews with service providers [14, 15]. The study aimed to examine client and provider perspectives on the acceptability of MOUD among PWID in Uganda.

### Study site

This study was conducted at the MOUD centre inside Butabika National Referral Mental Hospital, which serves the greater Kampala metropolitan area in Mukono and Wakiso districts. We chose a location in an urban area that is estimated to house approximately 8,000 PWID. The MOUD centre offers daily outpatient oral methadone and buprenorphine to participants. At the time of the study, over 460 individuals enrolled, clinic attendance rates range from 60–75% daily, with up to five new participants enrolled daily. The multidisciplinary team at the clinic includes psychologists, medical officers, social workers, psychiatric nurses, clinic officers, pharmacists, counsellors, peers, and security guards, all under the oversight of a psychiatrist.

### Study population and participant selection

The study population included PWID (some enrolled and others not enrolled in the MOUD program) and MOUD service providers. Enrolled PWID were recruited from among clients on MOUD during the clinic day, working closely with peer-educators. Peer educators approached all clients at the triage point and provided information to them about the study. Non-enrolled participants PWID were recruited through the community drop-in centres; that is, where PWID frequent, socialise, and often get information about their health and referral. Eligible participants were adults aged 18 years or older with a history of opioid injection within the six months preceding the study. All participants gave informed consent before participating in interviews. Appointments were scheduled with potential participants for in-depth interviews and key informant interviews. In line with the principle of data saturation [16], we conducted interviews with 20 PWID (10 enrolled and 10 not enrolled) and 10 service providers, including social workers (3), psychiatric nurses (2), a pharmacist (1), clinicians (2), and peer workers (2) (Table 1).

### Data collection procedures and methods

Data were collected using structured in-depth (IDI) and key informant interview(KII) guides developed for PWID and MOUD service providers, respectively. Interviews were conducted at the clinic or drop-in centre in the community, in either English or Luganda, the most widely spoken local language in the Kampala Metropolitan Area.

Data collection focused on PWID and service providers’ perceptions of MOUD acceptability as an effective treatment for PWID, including barriers to its use. Acceptability’ was defined as the perceived appropriateness, satisfaction, cultural fit, and emotional or cognitive response to an intervention by recipients and providers, shaping engagement, uptake, and sustainability[17–20]. Research assistants informed participants about the study before each interview to ensure informed understanding The topic guides were developed based on selected theoretical constructs from the theoretical framework of acceptability (TFA) [20]. Additionally, demographic information was collected before the interview. Each interview lasted approximately one hour and was audio-recorded. Participants’ identities were kept anonymous. The period for data collection and interpretation was two months.

### Data analysis

Data were organised and transcribed verbatim, and each transcript was saved using a corresponding unique identifier to the audio. We applied both deductive and inductive coding to identify themes related to perspectives, barriers, and acceptability [21] onto the theoretical framework of acceptability (TFA) [20]. A coding framework was designed based on four transcripts manually reviewed and coded to create the initial set of codes developed by MP, OS, and AT. All the transcripts were then uploaded into the ATLAS.ti v14 software [22] for analysis by MP. We employed thematic analysis, involving the identification of explicit and implicit ideas in the data and grouping these ideas as themes across the dataset. Illustrative quotations were selected to support each emergent theme in the results. We applied the consolidated criteria for reporting qualitative research (COREQ) checklist to the presentation of findings [23].

## RESULTS

### Characteristics of the participants

Of the 20 PWID, 16 were male, aged 22–50 years. Most enrolled participants had attained secondary education, whereas non-enrolled participants had at most primary education levels. Many lived over 5 km from the clinic. Long-term injecting histories were more common among enrolled clients. Most service providers had attained at least secondary education or higher, and had an average of 3 years’ experience of delivering MOUD. (Table 2).

### Mapping MOUD Acceptability Across TFA Constructs

Using TFA-guided thematic analysis, the study identified key constructs shaping the acceptability of MOUD among PWID in Uganda. Affective attitude was reflected in clients’ emotional appreciation of MOUD as a safer, life-restoring option. Perceived effectiveness emerged through narratives of improved health and social reintegration. Self-efficacy was linked to motivation for change and confidence in recovery. Ethicality was supported by cultural and moral alignment with MOUD. Intervention coherence was strengthened by psychosocial support but weakened by myths and misinformation. Opportunity cost highlighted the tension between daily clinic attendance and income-generating needs. Burden encompassed transport challenges, stigma, and fear of arrest. These themes, mapped across the TFA domains, revealed that while MOUD was widely valued, its uptake was hindered by structural and social barriers (Table 3).

#### MOUD as a safe treatment: Reclaiming life through supervised therapy

Participants consistently articulated MOUD as a safer treatment for injecting drug use disorder compare to the alternative treatment such as spiritual/social confinement, and abstinence-based rehabilitation. This view was stronger among those enrolled, who attributed their recovery to the program’s structured and comprehensive care. One 47-year-old woman shared reflected.

“… I have not used in over two years now. I got employed and I do not have any thoughts of any kind whatsoever of even smoking one line…….MAT… what can I say? It is the beginning of a new life.”

(IDI, female, enrolled).

Even among individuals not yet enrolled, there was a clear sense of anticipation and hope to engage with the program. One participant expressed:

I want it too, and as soon as you tell me to start taking methadone, ……. I am ready to start ……. because I want to get better.

(IDI, 30-year-old male, not enrolled)

Such narratives reveal strong confidence in MOUD’s effectiveness and reinforce the urgency of scaling up its availability and reach.

#### Good health outcome: The Physical and Psychological Rebirth

The Client and provider narratives converged around a shared observation that MOUD supports recovery beyond stopping drug use. Participants cited improvements in physical appearance, mental clarity, emotional stability, and overall well-being. These transformations reflected not only physiological recovery but also a renewed sense of self after years of marginalisation. One participant who dropped out of treatment stated:

“The little time I spent there helped me a lot… I left some drugs. I experienced a difference …..and I now look different”

(IDI, female, not enrolled).

The providers echoed these MOUD associated changes as visible and compelling among enrolled clients. A pharmacist recounted:

“I have seen success stories. People are back to employment and they have been reunited with their families”

(KII, male pharmacist).

These narratives highlight MOUD’s role in recovery, restoring stability and enabling reintegration into family, work, and community life.

#### Desire to become a better person

For many enrolled clients, recovery was a personal journey toward self-improvement, not merely clinical stabilization. This internal motivation often reflected growing confidence in managing treatment and sustaining recovery, aligning with the TFA’s self-efficacy construct. One participant shared,

“I am confident because my life is now okay. I move very well, ……., I take my drug, they teach me …and I am seeing that my life is changing”

(IDI, male, enrolled).

These reflections demonstrated increasing self-efficacy, strengthening participants’ confidence in engaging with treatment and accepting MOUD as effective.

#### Cultural Alignment and Ethical Acceptance of MOUD

Aligned with the ethicality construct, participants viewed MOUD as consistent with their personal and social/religious values, which encourage engagement. As one participant explained:

“With my religious beliefs, there is no problem. …. they are happy when they treat a person and that person gets fine,”

(IDI, male, enrolled).

Service providers echoed the importance of individualized person-centered treatment plans. As one noted:

“Everyone’s goal here is recovery. Some find it difficult [to retire from drugs], while others find it fair, which is why we [health workers] tailor a treatment plan for each person,”

(KII, male, clinical psychiatrist).

These insights underscore MOUD’s perceived moral legitimacy as a facilitator of acceptability.

#### Supportive systems and enabling resources for MOUD

Social support, particularly from family members, emerged as a crucial pillar in sustaining recovery. Supportive families enhanced confidence and accountability, reinforcing continued engagement in treatment. Knowing one was being supported strengthened participants’ resilience. Participants described family members as sources of emotional encouragement, support, and moral grounding. One enrolled participant shared,

“My father understands addiction very well and supported me throughout my recovery journey,”

(IDI, female, enrolled).

Beyond family, participants highly valued the clinic’s comprehensive, person-centered approach to care. MOUD was perceived as part of an integrated package of services that addressed broader health and psychosocial needs—well beyond the mere administration of medication. Participants reported receiving care for co-morbid conditions, individual counselling, peer support, and assistance with family reconciliation.. One participant described the clinic’s attentiveness to clients’ broader needs:

“…when you’re sick, they help with medication…or refer you elsewhere. And they can give you directions,”

(IDI, female, enrolled).

Another participant added:

“they even give us psychological therapy. And our doctors continue having personal dialogue with us. They continue sensitising us about the drugs and mixing that up with methadone,”

(IDI, male, enrolled).

Participants viewed these services as vital to their recovery, with regular provider interaction, counseling, and broader support encouraging continued engagement in the program.

### Barriers to accessing MOUD

#### Opportunity cost for MOUD: When Recovery Competes with Survival

The construct of opportunity cost emerged prominently in participants’ accounts. For many, daily clinic attendance was logistically demanding and financially burdensome. Participants explained how commuting daily competed with essential activities like securing food, and income, and fulfilling family responsibilities. Some suggested introducing multi-day take-home doses to alleviate clinic visits. A non-enrolled participant said’

“I can’t afford going to the clinic daily, maybe if they could give me (MAT) for one week… the guards at the gate check you and remove your jacket before entry,”

(IDI, male, not enrolled).

Participants often faced difficult choices between accessing treatment and addressing immediate survival needs. Some relocated closer to clinics to minimize transport costs; however, this led to significant social and economic trade-offs, including family separation and disruption of income-generating activities. A service provider confirmed:

“….most of them have shifted from far places where they were staying to come nearer to be able to access the MOUD clinic,”

(KII, female clinical psychiatrist).

#### Perceived effectiveness of MOUD and Alternative Healing Practices

Perceived effectiveness emerged as a central factor shaping participants’ attitudes towards MOUD. Some participants, particularly those not enrolled, doubted methadone’s potency compared to heroin, fuelling treatment hesitancy. One participant recounted,

“They told me to quit drugs, which I did, but like I said, I am still taking alcohol”

(IDI, male, not enrolled).

Strict clinic enrolment criteria requiring injection evidence further complicated treatment access, as one enrolled participant recounted:

“They sent me away because of no signs of injection….. I started injecting then I went back to the clinic with visible marks and then I was given “MAT”,””

IDI, male, enrolled).

Both enrolled and non-enrolled participants described widespread reliance on traditional healers, and spiritual or private abstinence-based treatments, either voluntary or coerced. A non-enrolled participant shared:

“Sometimes, I go to the pastor and … I stay there for some days not taking drugs, but then you feel bad again and escape back to the ghetto,”

(IDI, female, single, not enrolled).

Other narratives underscore treatment pathways influenced by traditional beliefs. Even among those later enrolled in MOUD, traumatic abstinence-based treatment experiences often preceded MOUD. for instance, an enrolled participant recounted how his family responded to his drug use problem:

“Before my family took me to the pastor and to some shrine in Mukono district, they tied me up with ropes and confined me to the shrine for one month,”

(IDI, male, enrolled).

#### Disruption of social norms and peer resistance

While MOUD was generally perceived positively, some participants described it as disrupting established social norms within drug-using communities. These norms emphasized mutual dependence and collective drug use, with needle sharing symbolizing trust and solidarity. Consequently, MOUD enrolment, created tension, provoking suspicion within PWID communities toward individuals who ceased practices like sharing injection equipment. One non-enrolled participant explained,

“In the ghetto, sharing needles is a sign of trust. MOUD makes people suspicious of those who stop sharing,”

(IDI, 38-year-old male, not enrolled).

Participants also expressed concerns about social isolation and loss of peer relationships when they decide to start on MOUD. as stated by a participant:

“…I will miss some of the good friends. Once I leave, I will never come back to the ghetto,”

(IDI, male, not enrolled).

These results highlight a critical barrier to MOUD acceptance arising from established social practices within drug-using communities, that conflicts with medical treatment

#### Structural Barriers: Transport, law enforcement, and gendered vulnerabilities

Structural barriers, including transport difficulties, fear of arrest by law enforcers, and gender-related constraints, significantly heightened participants’ perceived burden of accessing MOUD services. Participants described financial and logistical strains associated with daily commutes, particularly affecting those from far areas and especially women balancing household obligations with treatment needs.

A non-enrolled female participant shared,

“I cannot afford the daily commute to the clinic. I must prioritise food and rent over transportation costs,”

IDI, female, not enrolled.

Service providers confirmed these assertions as a nurse explained:

“..there are some who say, I have a challenge maybe with transport because they come from very far, …..and so, someone tells you; ‘I have failed to get transport’,”

(KII, Nurse).

Further fear of arrest emerged as a significant deterrent to clinic attendance. Participants expressed anxiety about being identified as drug users while visiting treatment centres, due to the criminalisation of drug use in Uganda. A participant recalled,

“I was there for 3 days… I got arrested, but I am going to resume from where I ended”

(IDI, 30-year-old male), emphasizing logistical and safety barriers restricting MOUD participation.

#### Stigma and discrimination

Stigma (both societal and institutional), emerged as a pervasive barrier to MOUD acceptability and access. Participants commonly reported being viewed as morally deficient, or weak, which discouraged help-seeking and reduced willingness to engage openly with services. One male participant explained.

“People look down on us because we are addicts. They think we are weak. You fear to seek help when you know you will be judged,”

(IDI, male, not enrolled).

Participants described being stigmatised in community settings, where drug use was equated with failure and worthlessness. Female participants reported heightened scrutiny due to gendered expectations. One woman reflected,

“As a woman, I face much stigma as an addict, ……. It is isolating,”

(IDI, female, not enrolled).

Discrimination within healthcare care settings was also common. Some participants described being dismissed or mistrusted by health workers. One enrolled participant stated,

“I have been turned away from clinics…. Doctors treat me differently; like I am not deserving of care, ……. and makes me reluctant to seek help,”

(IDI, 50-year-old male, enrolled).

For some, receiving services at a psychiatric facility further exacerbated their sense of stigma, due to prevailing beliefs about mental illness. A service provider remarked:

“*Some people fear coming to a mental hospital …….they are stigmatised for coming to ‘a hospital for the mentally ill’,”*

(KII, Male psychiatrist).

#### Myths and misconceptions: Undermining trust in treatment

Participants held misconceptions regarding MOUD, particularly that methadone causes impotence or is itself addictive. Some non-enrolled individuals doubted its safety, viewing it as equally or more harmful than the drugs it replaces. One non-enrolled participant explained:

“When health workers come here, they don’t tell us that MOUD causes impotence …… they only say get enrolled,.. yet it is also an addiction drug like heroin,”

(IDI, male, not enrolled).

Concerns also centered on whether MOUD offers real health benefits or simply substitutes one drug for another, especially regarding its effects on sexual function and fertility. These misconceptions were common among PWID networks and community discussions. Another participant recounted:

“I would want to be helped but again I am told that methadone is also a drug and not medicine. Is it true?”

(IDI, female, not enrolled).

Service providers acknowledged the widespread myths and their role in shaping community attitudes toward MOUD. One service provider explained,

“We need to engage with the communities to dispel myths ……..fostering dialogue and understanding, we can reduce stigma and create a more supportive environment for individuals in recovery,”

(KII, male nurse).

Such misconceptions present critical barriers to MOUD uptake, underscoring the need for targeted education and community engagement

## DISCUSSION

Effective implementation of health programs hinges on the perceived acceptability of the intervention by both providers and recipients[24–26]. This study explored both the providers’ and recipients’ perspectives on the acceptability of MOUD in Uganda. The TFA was applied to examine the emotional, social, and structural dimensions of treatment engagement [20]. MOUD was widely viewed as life-restoring and acceptable among participants. Acceptability was underpinned by intersecting affective, cognitive, and ethical constructs, particularly emotional connection, perceived effectiveness, cultural alignment, and confidence in the ability to adhere to treatment. However, these enablers were also moderated by significant social and structural barriers that shaped who accessed, continued, or disengaged from care.

Participants consistently described MOUD as transformative, safe, and restorative. Methadone was described not only as a safer alternative to injecting drugs or abstinence-based rehabilitation but also as a catalyst for reclaiming stability, health, and dignity. This strong emotional connection, consistent with the affective attitude construct of the TFA, positioned MOUD as a vehicle for rebuilding self-worth, repairing family ties, and restoring social identity. Clients described their experience with the program as life-saving, and for many, it marked a turning point in their trajectory.

Affective engagement was closely tied to the holistic, person-centered nature of service delivery. Participants valued the integration of methadone with ancillary services such as counselling, treatment of comorbidities, psychosocial support, and peer education offered through the clinic and community-based partner, the Uganda Harm Reduction Network [27]. These dimensions created a sense of respect, trust, and belonging, which strengthened client satisfaction and commitment to the program. Such integrative models have been shown to enhance treatment uptake and retention [28, 29]. Service providers played a central role in facilitating this experience. Their nonjudgmental, person-centered approach to meeting each client’s needs generated strong emotional bonds with the program. Many providers also emphasised how respectful and consistent relationships provide therapeutic benefits that boost clients’ motivation and retention.

Positive emotional responses to MOUD extended beyond those already enrolled. Many non-enrolled participants expressed strong affective openness to treatment, associating it with the hope of becoming better people and rejoining mainstream social life. This emotional readiness, despite barriers such as stigma, misinformation, or lack of access, suggests latent demand and high perceived value. Comparable findings were reported in Ukraine and the USA, where willingness to enrol was shaped by exposure to peer support and counselling despite persistent structural challenges[30, 31].

Acceptability was further reinforced through perceived effectiveness. Participants consistently reported improvements in physical health, psychological well-being, and social functioning. Beyond symptom control, MOUD was experienced as a comprehensive intervention that addressed both clinical and psychosocial needs. Similar patterns have been observed globally, where MOUD acceptability is closely tied to the breadth of services available and the responsiveness of care systems[2, 32–34]. Providers confirmed these recipient testaments, sharing accounts of clients who resumed employment, reconciled with family, or experienced mental health improvements. Such perspectives were a reinforcement of the perceived value of the intervention.

Findings also highlight the role of self-efficacy in the acceptability of health service delivery. Enrolled participants expressed confidence in adhering to treatment and sustaining recovery when supported by providers. For some, this self-belief was grounded in a moral or religious framework, which positioned MOUD as a legitimate and even virtuous path to transformation. This alignment with personal values, consistent with the TFA’s ethicality construct, was reinforced by provider respect and community support. Evidence from other contexts supports this interpretation. For example, studies by Price and colleagues show that emotionally supportive, body-aware interventions foster personal agency and improve treatment retention by helping clients reconnect with their sense of control and self-worth [35]. Similar interpretations have also been documented in East Africa and elsewhere, where spiritual and cultural narratives are used to contextualise the treatment of substance use as a socially redemptive practice [36–39].

Despite these positive perceptions, participants described a range of barriers that limited MOUD acceptability. These barriers were deeply structural and often experienced as burdens, corresponding with the TFA’s opportunity cost and burden constructs. Daily attendance requirements created logistical and financial strain, especially for clients balancing survival needs such as food, shelter, and income generation. Long commutes, transport costs, and lack of take-home dosing compounded these challenges. These opportunity costs have been well documented in low-resource contexts where inflexible service delivery deters those most in need [40–42].

Stigma also emerged as a profound constraint, intersecting with gender, socioeconomic status, and healthcare interactions. Participants described being judged or excluded by family members, peers, and even health workers. Women experienced a dual stigma because their drug use broke gendered social norms as well as societal expectations around drug use. Similar patterns have been extensively documented in the literature, where stigma contributes to concealment, treatment avoidance, and poor health outcomes, particularly when compounded by gender-related vulnerabilities[41, 43, 44].

Peer dynamics within drug-using communities also influenced acceptability. Some participants viewed MOUD as undermining communal norms like drug sharing, which was regarded as a symbol of trust and solidarity. The PWID who ceased injecting or distanced themselves from the group risked suspicion or social exclusion. Bryant et al. (2010), and other researchers from Europe and Sub-Saharan Africa, observed that MOUD can fracture peer ties and introduce new vulnerabilities [45–47].

Persistent misinformation on MOUD, fear of arrest, and restrictive clinic practices collectively shaped how participants navigated treatment acceptability. Rather than engaging with MOUD, many were diverted, voluntarily or involuntarily into abstinence-based care offered by spiritual, traditional, or private providers. These alternatives were often perceived as safer or more acceptable, yet they remain unregulated and disconnected from evidence-based harm reduction practices [2, 48]. Similar patterns have been reported in Vietnam, Kenya, and Nigeria, where structural exclusions from MOUD push clients toward informal systems that may delay or deny effective care [49, 50].

This diversion to alternative non-standard treatment options, reflects more than gaps in access; it signals a deeper erosion of community trust in formal MOUD services. Criminalisation, widespread misinformation, and punitive drug policies contribute to an environment in which treatment is viewed as unsafe, unfamiliar, or morally objectionable. Global evidence shows that legal and regulatory barriers, including restrictions on who can provide MOUD and criminalisation of drug use, continue to impede scale-up and service delivery [51–53]. At the clinic, strict enrolment criteria and inflexible dosing requirements further discourage participation. Together, these structural and procedural challenges make the unregulated traditional, spiritual healing or private rehabilitation, appear more accessible and culturally acceptable, even when they offer limited clinical and social benefit. Addressing these barriers requires public education and community collaboration to enhance understanding of MOUD. Revising enrolment policies and minimising security-related barriers at clinics are equally essential to improving access and engagement.

Collectively, these findings affirm that the acceptability of MOUD operates on multiple levels. Emotional readiness, cultural alignment, and perceived effectiveness powerfully influence how PWID accept to treatment. However, these positive experiences are constrained by the realities of poverty, stigma, misinformation, and criminalisation. Efforts to scale up MOUD in Uganda should go beyond pharmacological delivery to include flexible service models, provider training, peer engagement, and community sensitisation.

## STRENGTHS

This study offers a pragmatic examination of the acceptability of medications for opioid use disorder in Uganda, contributing valuable insights from both service recipients and providers. A key strength lies in its inclusion of diverse participant categories, capturing multiple viewpoints from individuals currently enrolled in MOUD programs, those not enrolled, and healthcare providers. This triangulation of viewpoints enriched the data and facilitated a more nuanced understanding of the contextual factors influencing acceptability. The application of the TFA provided a robust analytical lens to assess emotional, cognitive, ethical, and structural dimensions of MOUD uptake.

The study followed rigorous qualitative procedures, employing skilled interviewers for data collection, and a systematic coding process for analysis using ATLAS.ti software. Reflexivity was actively maintained through peer debriefing, along with an audit trail, and recording when thematic saturation was achieved. Furthermore, the study contributes novel insights into the interactions between formal MOUD services and the broader ecosystem of addiction treatment, which includes traditional, spiritual, and private rehabilitation settings. These findings suggest meaningful implications for scaling up MOUD services across Uganda’s pluralistic health system.

## LIMITATIONS

Despite the valuable insights, some limitations exist. The study was conducted in an urban setting, which may not represent the experiences of PWID in rural areas where health infrastructure, stigma, and access barriers may differ. The exclusion of key informants such as private rehabilitation providers, spiritual and traditional healers, and law enforcement personnel limited the breadth of perspectives captured. Anecdotal reports indicate that some police officers are increasingly reluctant to detain individuals who use drugs due to withdrawal crisis risks, opting instead to refer them for treatment. Similarly, private and faith-based rehabilitation facilities remain prominent sources of care for individuals with substance use disorders in Uganda. Their omission restricts the analysis of how informal systems influence client perceptions and pathways into or away from MOUD.

Another limitation relates to the reliance on self-reported data may introduce potential recall and social desirability biases, given the criminalised nature of drug use in Uganda. It is also possible that participants may withhold information related to law enforcement encounters or negative experiences within the health system. Nonetheless, the study offers a strong foundation for future research and policy engagement to enhance the accessibility and acceptability of MOUD services in Uganda and similar low-resource settings.

## CONCLUSION

MOUD was highly acceptable among PWID and service providers, with participants valuing its effectiveness and potential to restore health and stability. However, transport burdens, daily attendance requirements, opportunity costs, stigma, misinformation, and restrictive enrolment criteria limited access to MOUD. These limitations result in the inability or delayed access to appropriate care. To support scale-up, MOUD programs should adopt more flexible service models and simplify enrolment. Additionally, targeted community sensitisation and coordinated engagement with private, religious, and traditional actors, alongside law enforcement, are essential to promote early and sustained treatment uptake.

## Figures and Tables

**Table 1:** Distribution of the research participants

Category of participant	Number
**PWID**	**20**
Not enrolled on MOUD	10
Currently enrolled on MOUD	10
**MOUD service providers**	**10**
Social worker/counsellor	2
Psychiatric nurse	3
Pharmacist/ Assistant	2
Clinician/Medical/clinical officer	3

**Table 2:** Characteristics of study participants

Variable	Participants not enrolled	Enrolled participants	Service providers
Sex Female	2	2	7
Age ->30years	8	7	7
Marital status- Married	4	2	5
Education level			
Secondary/higher	3	10	10
Primary level	4	0	0
Employment status: Employed	5	5	10
Staying >5km from MOUD clinic	10	5	NA
Duration of injecting drugs (>5years)	5	7	NA
Duration of services provision (>2years)	NA	NA	3

NA = Not applicable

**Table 3:** TFA-guided thematic analysis diagram with linked constructs, themes, and participant quotes

TFA Construct	Themes Emerging(illustrative quotes)
Affective Attitude	**MOUD as a Safe Treatment** *“MOUD viewed as safer than injecting or traditional healing.’, ‘Participants credited it with life transformation and sobriety.”*
Perceived Effectiveness	**Good Health Outcomes** *“Clients reported improved physical appearance and well-being.’, ‘Providers observed reintegration into family and employment.”*
Self-Efficacy	**Desire to Become a Better Person** *“Participants showed strong motivation to change.’, ‘Enrolled clients expressed growing confidence in their recovery.”*
Ethicality	**Cultural Alignment and Ethical Acceptance of MOUD** *“MOUD aligned with participants*’ *religious and moral values.’, ‘Service providers tailored treatment to individual contexts.”*
Affective Attitude & Intervention Coherence	**Supportive Systems and Enabling Resources** *“Family support seen as critical to recovery success.’, ‘Clinic provided holistic services beyond methadone dispensing”*
Opportunity Cost	**Opportunity Costs** *“Daily clinic visits competed with food and income needs.’, ‘Some clients suggested multi-day take-home doses”*
Perceived Effectiveness	**Perceived Limitations of MOUD and Alternative Healing Practices** *“Methadone viewed by some as less potent than heroin.’, ‘Traditional and spiritual healing often sought first.”*
Burden (social cost)	**Disruption of Social Norms and Peer Resistance** *“Drug-sharing seen as a norm of trust among peers.’, ‘MOUD enrolment led to suspicion and social isolation.”*
Burden	**Structural Barriers** *“High transport costs and long distances limited access.’, ‘Fear of arrest discouraged clinic visits for some clients/’*
Affective Attitude & Ethicality	**Stigma and Discrimination** *“Clients feared judgement and moral condemnation from society.’, ‘Women faced added stigma tied to gender roles.”*
Intervention Coherence	**Myths and Misconceptions** *“Some believed methadone causes impotence or is addictive.’, ‘Providers used testimonies to dispel misinformation.”*

Figure xx: TFA-guided thematic analysis diagram with linked constructs, themes, and participant quotes

## Data Availability

De-identified data and analysed data for this manuscript is available from the corresponding author upon reasonable request by the corresponding

## Abbreviations

MOUD: Medication for opioid use disorder
OUD: opioid use disorder
PWID: people who inject drugs
PWUD: people who use drugs
UHRN: Uganda Harm Reduction Network

## References

[1] UNODC. World Drug Report (United Nations publication, 2023). 2023.

[2] WHO U. Technical Guide for countries to set targets for universal access to HIV prevention, treatment and care for injecting drug users. 2009.

[3] Amura CR, Sorrell TR, Weber M, Alvarez A, Beste N, Hollins U Outcomes from the medication assisted treatment pilot program for adults with opioid use disorders in rural Colorado. Substance Abuse Treatment, Prevention, and Policy. 2022;17:1–11. 34980179 10.1186/s13011-021-00424-4 PMC8722086

[4] Saloner B, Krawczyk N, Solomon K, Allen ST, Morris M, Haney K, Experiences with substance use disorder treatment during the COVID-19 pandemic: Findings from a multistate survey. Int J Drug Policy. 2022;101:103537. 34871945 10.1016/j.drugpo.2021.103537 PMC8602971

[5] MacArthur GJ, Minozzi S, Martin N, Vickerman P, Deren S, Bruneau J Opiate substitution treatment and HIV transmission in people who inject drugs: systematic review and meta-analysis. BMJ. 2012;345. 10.1136/bmj.e5945 PMC3489107 23038795

[6] Carter J, Li Z, Chen H, Greiner M, Bush C, Bhattacharya D, Low barrier medication for opioid use disorder at a federally qualified health center: a retrospective cohort study. Addict Sci Clin Pract. 2022;17(1):60. 36335381 10.1186/s13722-022-00342-1 PMC9636799

[7] Abbo C. Traditional Healers and Mental Health Problems in Uganda. LAP Lambert Academic Publishing; 2010.

[8] Johnson LR, Chin EG, Kajumba M, Kizito S, Bangirana P. Views on depression from traditional healing and psychiatry clinics in Uganda: Perspectives from patients and their providers. J Cross-Cult Psychol. 2017;48(2):243–61.

[9] Ndetei DM. Traditional healers and provision of mental health services in cosmopolitan informal settlements in Nairobi, Kenya. Afr J psychiatry. 2013;16(2):134–40. 10.4314/ajpsy.v16i2.17 23595533

[10] Galabuzi C, Agea J, Fungo B, Kamoga R. Traditional medicine as an alternative form of health care system: a preliminary case study of Nangabo sub-county, central Uganda. Afr J Tradit Complement Altern Med. 2010;7(1). 10.4314/ajtcam.v7i1.57224 PMC3005386 21304607

[11] Berhe KT, Gesesew HA, Ward PR. Traditional healing practices, factors influencing to access the practices and its complementary effect on mental health in sub-Saharan Africa: a systematic review. BMJ open. 2024;14(9):e083004. 10.1136/bmjopen-2023-083004 PMC11429370 39322598

[12] Ndetei D, Musyimi C, Nandoya E, Matoke L, Mutiso V. Working with traditional healers to reduce the mental health treatment gap in low-and middle-income countries. Oxford textbook of public mental health. 2018:559–66.

[13] Mudiope P, Mutamba BB, Komuhangi L, Nangendo J, Alamo S, Mathers B, Retention of people who inject drugs enrolled in a ‘medications for opioid use disorder’(MOUD) programme in Uganda. Addict Sci Clin Pract. 2024;19(1):39. 38750568 10.1186/s13722-024-00468-4 PMC11094991

[14] Creswell JW. A concise introduction to mixed methods research. SAGE; 2021.

[15] Yüksel P, Yıldırım S. Theoretical frameworks, methods, and procedures for conducting phenomenological studies in educational settings. Turkish online J qualitative Inq. 2015;6(1):1–20.

[16] Francis JJ, Johnston M, Robertson C, Glidewell L, Entwistle V, Eccles MP, What is an adequate sample size? Operationalising data saturation for theory-based interview studies. Psychol health. 2010;25(10):1229–45. 20204937 10.1080/08870440903194015

[17] Bowen DJ, Kreuter M, Spring B, Cofta-Woerpel L, Linnan L, Weiner D, How we design feasibility studies. Am J Prev Med. 2009;36(5):452–7. 19362699 10.1016/j.amepre.2009.02.002 PMC2859314

[18] Organization WH. Quality of care: a process for making strategic choices in health systems. Quality of care: a process for making strategic choices in health systems 2006.

[19] Proctor E, Silmere H, Raghavan R, Hovmand P, Aarons G, Bunger A, Outcomes for implementation research: conceptual distinctions, measurement challenges, and research agenda. Adm policy mental health mental health Serv Res. 2011;38:65–76. 10.1007/s10488-010-0319-7 PMC3068522 20957426

[20] Sekhon M, Cartwright M, Francis JJ. Acceptability of healthcare interventions: an overview of reviews and development of a theoretical framework. BMC Health Serv Res. 2017;17:1–13. 28126032 10.1186/s12913-017-2031-8 PMC5267473

[21] Braun V, Clarke V. Using thematic analysis in psychology. Qualitative Res Psychol. 2006;3(2):77–101.

[22] Friese S. Qualitative data analysis with ATLAS. ti. Qualitative data analysis with ATLAS ti. 2019:1–344.

[23] Tong A, Sainsbury P, Craig J. Consolidated criteria for reporting qualitative research (COREQ): a 32-item checklist for interviews and focus groups. Int J Qual Health Care. 2007;19(6):349–57. 17872937 10.1093/intqhc/mzm042

[24] Diepeveen S, Ling T, Suhrcke M, Roland M, Marteau TM. Public acceptability of government intervention to change health-related behaviours: a systematic review and narrative synthesis. BMC Public Health. 2013;13:1–11. 23947336 10.1186/1471-2458-13-756 PMC3765153

[25] Mrc U. Developing and evaluating complex interventions: new guidance. London: Medical Research Council; 2008.

[26] Stok FM, De Ridder DT, De Vet E, Nureeva L, Luszczynska A, Wardle J, Hungry for an intervention? Adolescents’ ratings of acceptability of eating-related intervention strategies. BMC Public Health. 2015;16:1–8. 10.1186/s12889-015-2665-6 PMC4700578 26729328

[27] UHRN. Uganda Harm Reduction Network (UHRN), Retrieved. April 13, 2025, from https://ugandaharmreduction.org/ (no title) + 2 2025 [.

[28] Samet JH, Botticelli M, Bharel M. Methadone in primary care—one small step for Congress, one giant leap for addiction treatment. N Engl J Med. 2018;379(1):7–8. 29972744 10.1056/NEJMp1803982

[29] Timko C, Schultz NR, Cucciare MA, Vittorio L, Garrison-Diehn C. Retention in medication-assisted treatment for opiate dependence: a systematic review. J Addict Dis. 2016;35(1):22–35. 26467975 10.1080/10550887.2016.1100960 PMC6542472

[30] Polonsky M, Rozanova J, Azbel L, Bachireddy C, Izenberg J, Kiriazova T, Attitudes toward addiction, methadone treatment, and recovery among HIV-infected Ukrainian prisoners who inject drugs: incarceration effects and exploration of mediators. AIDS Behav. 2016;20:2950–60. 27011378 10.1007/s10461-016-1375-0 PMC5035551

[31] Fox AD, Riback L, Perez-Correa A, Ohlendorf E, Ghiroli M, Behrends CN, High interest in Injectable Opioid Agonist Treatment with Hydromorphone among Urban Syringe Service Program participants. Subst Use Addict J. 2024;45(1):44–53. 10.1177/29767342231210552 38258851

[32] Hagedorn H, Dieperink E, Dingmann D, Durfee J, Ho SB, Isenhart C, Integrating hepatitis prevention services into a substance use disorder clinic. J Subst Abuse Treat. 2007;32(4):391–8. 17481462 10.1016/j.jsat.2006.10.004

[33] Organization WH. Consolidated guidelines on HIV prevention, testing, treatment, service delivery and monitoring: recommendations for a public health approach. World Health Organization; 2021. 34370423

[34] Rhodes T. The becoming of methadone in Kenya: how an intervention’s implementation constitutes recovery potential. Soc Sci Med. 2018;201:71–9. 29455053 10.1016/j.socscimed.2018.02.007 PMC5922264

[35] Price CJ, Hooven C. Interoceptive awareness skills for emotion regulation: Theory and approach of mindful awareness in body-oriented therapy (MABT). Front Psychol. 2018;9:798. 29892247 10.3389/fpsyg.2018.00798 PMC5985305

[36] Bryson EO, Boxhorn CE. The Opioid Epidemic: Origins, Current State and Potential Solutions. Cambridge University Press; 2023.

[37] Mlunde LB, Hirschhorn LR, Nyblade L, Rothrock NE, Mbugi EV, Moskowitz JT, Translation and cultural adaptation of drug use stigma and HIV stigma measures among people who use drugs in Tanzania. PLoS ONE. 2023;18(10):e0292642. 37856437 10.1371/journal.pone.0292642 PMC10586607

[38] Obare EOE. The culture of drug abuse and substance use as a determinant of health outcomes among students in Kenya public universities. Afr J Alcohol Drug Abuse. 2022;7(2):54–65.

[39] Ludwig-Barron NT, Guthrie BL, Mbogo L, Bukusi D, Sinkele W, Gitau E, Barriers and facilitators of HIV and hepatitis C care among people who inject drugs in Nairobi, Kenya: a qualitative study with peer educators. Harm Reduct J. 2021;18(1):133. 34922548 10.1186/s12954-021-00580-7 PMC8684158

[40] Aea Dutta. Strengthening opioid substitution therapy access in sub-Saharan Africa. BMC Health Serv Res. 2022;22(834):834.35765059

[41] Wolf HT, Halpern-Felsher BL, Bukusi EA, Agot KE, Cohen CR, Auerswald CL. It is all about the fear of being discriminated [against]… the person suffering from HIV will not be accepted: a qualitative study exploring the reasons for loss to follow-up among HIV-positive youth in Kisumu, Kenya. BMC public health. 2014;14:1–11. 25377362 10.1186/1471-2458-14-1154 PMC4232620

[42] Morton CM. Community social deprivation and availability of substance use treatment and mutual aid recovery groups. Substance abuse treatment. Prev policy. 2019;14:1–5. 10.1186/s13011-019-0221-6 PMC6701142 31426822

[43] Baus AD, Carter M, Boyd J, McMullen E, Bennett T, Persily A, A Better life: factors that help and hinder entry and retention in MAT from the perspective of people in recovery. J Appalach Health. 2023;5(1):72. 38023116 10.13023/jah.0501.06 PMC10629892

[44] Nieburg P, Carty L. HIV prevention among injection drug users in Kenya and Tanzania. Centre for Strategic and International Studies; 2011.

[45] Bryant J, Treloar C, Fraser S. Methadone maintenance therapy and social control: The making of a good addict. Int J Drug Policy. 2010;21(6):463–9.

[46] Knight D, Nkya IH, West NS, Yang C, Kidorf M, Latkin C, Economic, social, and clinic influences on opioid treatment program retention in Dar es Salaam, Tanzania: a qualitative study. Addict Sci Clin Pract. 2023;18(1):19. 36973794 10.1186/s13722-023-00374-1 PMC10042396

[47] Otiashvili D, Kirtadze I, O’Grady KE, Zule W, Krupitsky E, Wechsberg WM, Access to treatment for substance-using women in the Republic of Georgia: socio-cultural and structural barriers. Int J Drug Policy. 2013;24(6):566–72. 23756037 10.1016/j.drugpo.2013.05.004 PMC3797849

[48] NDA. Professional Guidelines for Regulation of Local Traditional/Herbal Medicines for Human and Veterinary Use in Uganda, Doc. No.7 DAR/GDL/033. In: National, Drug, Authority, editors. 2021.

[49] Kiburi SK, Mwangi J, Maina G. Exploring the experiences of clients receiving opioid use disorder treatment at a methadone clinic in Kenya: a qualitative study. Addict Sci Clin Pract. 2022;17(1):71. 36510246 10.1186/s13722-022-00352-z PMC9742652

[50] Kamarulzaman A, Reid SE, Schwitters A, Wiessing L, El-Bassel N, Dolan K, Criminalization of drug use and barriers to harm reduction: Evidence from a global systematic review. Lancet. 2016;387(10026):1429–40.

[51] Manthey J, Benegal V, Kilian C, Mahadevan J, Mejía-Trujillo J, Morojele N International Perspectives on Stigma toward People with Substance Use Disorders. The Stigma of Substance Use Disorders. 2022:107.

[52] UNAIDS. HIV and people who use drugs: The global situation and responses. Joint United Nations Programme on HIV/AIDS.; 2022.

[53] Policy GCoD. Time to end the criminalization of people who use drugs. Geneva: Global Commission on Drug Policy; 2021.

